# Arthroscopic all-inside ACL reconstruction with anterolateral tendon fixation: a prospective cohort study on restoring rotational stability in high-grade tibial shift

**DOI:** 10.3389/fsurg.2025.1614925

**Published:** 2025-07-16

**Authors:** Haoran Gu, Aifang Niu, Jingrui Ji, Miao He, Ying Yang, Junxia Lu, Jianghong Lv, Yongdong Yi, Hui Zhou, Wuping Zhou

**Affiliations:** ^1^ Traumatic Orthopedics, Army 947 Hospital, Kashgar, Xinjiang, China; ^2^ Health Service Training Center, Army 947 Hospital, Kashgar, Xinjiang, China; ^3^ Nursing Department, Air Force Medical University, Xi’an, Shanxi, China; ^4^ Medical Care Center, Army 947 Hospital, Kashgar, Xinjiang, China; ^5^ Department of Psychology and Neurology, Army 947 Hospital, Kashgar, Xinjiang, China

**Keywords:** anterior cruciate ligament reconstruction, all-inside technique, anterolateral augmentation, rotational stability, high-grade anterior tibial translation

## Abstract

**Aim:**

This study aimed to evaluate the early efficacy of combined all-inside anterior cruciate ligament (ACL) reconstruction and anterolateral tendon fixation in addressing knee instability associated with ACL rupture and high-grade anterior tibial translation (≥10 mm).

**Methods:**

In this prospective cohort study, 21 patients (16 men, 5 women; mean age: 27.4 ± 5.8 years) with ACL rupture and grade III anterior tibial displacement were selected from 90 consecutive cases treated between January 2019 and January 2020. All procedures were performed by a single surgeon using autologous semitendinosus tendon grafts (diameter: 8–9 mm). The all-inside ACL reconstruction was augmented with anterolateral tendon fixation utilizing the posterior fibers of the iliotibial band. Postoperative evaluations were conducted at immediate, 1-, 3-, 6-, and 12-month intervals and included: objective stability testing (Lachman and pivot-shift tests), functional outcome assessments (IKDC, Lysholm, and KOOS scores), and radiographic measurement of anterior tibial displacement.

**Results:**

All patients completed at least 12 months of follow-up, with no reported cases of recurrent instability. Immediate postoperative assessments revealed negative Lachman and pivot-shift tests in 100% of patients, indicating restored knee stability. At the 12-month follow-up, 90.5% (19/21) of patients maintained full stability, while the remaining two exhibited only grade I laxity, representing a significant improvement from preoperative grade III instability (*P* < 0.001). Functional outcomes also improved markedly, with mean IKDC scores increasing from 48.6 ± 10.3 preoperatively to 86.7 ± 3.6 at 12 months (*P* < 0.001), and Lysholm scores rising from 52.6 ± 12.4 to 89.6 ± 2.9 (*P* < 0.001). At final follow-up, 52.4% (11/21) of patients achieved “excellent” and 38.1% (8/21) “good” ratings on the Lysholm scale (*P* < 0.001 vs. baseline). Additionally, KOOS subscale analysis demonstrated significant pain reduction, with scores improving from 45.2 ± 9.1 preoperatively to 88.3 ± 4.7 postoperatively (*P* <  0.001).

**Conclusion:**

Combined all-inside ACL reconstruction and anterolateral tendon fixation could effectively restore anterior and rotational stability in knees with ACL rupture and high-grade tibial displacement. Early outcomes demonstrate promising functional recovery and objective stability at short-term follow-up, suggesting that this technique may offer biomechanical benefits for managing severe instability patterns. However, long-term studies are needed to confirm the durability of these results.

## Background

The anterior cruciate ligament (ACL) is located inside the knee joint, connecting the femur and tibia, and plays an important role in maintaining knee joint stability. ACL rupture severely limits knee joint function, and if not treated promptly and thoroughly, can lead to secondary damage to joint cartilage, meniscus, and other structures. According to epidemiological statistics, there are more than 100,000 ACL injury patients seeking medical treatment each year, with an incidence rate of 1/3,000, making it one of the most common sports injuries (1). With the in-depth exploration of ACL anatomy, surgical methods for anterior cruciate ligament reconstruction (ACLR) are constantly improving, and ACLR under arthroscopy has gradually become the mainstream treatment method (2). All inside technology-anterior cruciate ligament reconstruction (AIT-ACLR) establishes femoral and tibial tunnels through retrograde drilling of the tibia, not only preventing bone fragments from entering but also reducing the incidence of postoperative tunnel enlargement. Compared with conventional ACLR, it also has certain cosmetic effects (3). However, studies have found that simple ACLR surgery cannot effectively reduce rotational laxity, and postoperative knee joints often have residual rotational instability, with the main clinical feature being positive preoperative pivot shift test (grade II/III) (4–6). The anterolateral ligament (ALL) is an important structure for maintaining rotational stability of the knee joint. ACL rupture often complicates ALL injury, leading to anterior and rotational instability of the knee joint (6, 7). Relevant studies have found that the number of such patients is as high as 25% (8). Therefore, addressing the potential problem of knee joint rotational instability during ACLR surgery has gradually become a research focus in the field of sports medicine. This study aimed to improve the rotational stability of the knee joint in patients with ACL rupture combined with high-grade axial instability by performing AIT-ACLR and anterolateral tenodesis procedure (ALLT) simultaneously, and to summarize and analyze the early follow-up results.

## Materials and methods

### Patient data

A retrospective analysis was conducted on patients with ACL rupture combined with high-grade axial instability who were treated with AIT-ACLR + ALLT at the Department of Orthopedics, the 947th Hospital of the People's Liberation Army, China, from June 2021 to June 2022. All surgeries were performed by the same senior doctor. A total of 90 consecutive patients with ACL injuries were initially reviewed. After applying strict inclusion and exclusion criteria, 21 patients were ultimately included in the study. Among the included patients, there were 16 males and 5 females, with an average age of 27.4 years (range: 19–41 years). The average injury time before surgery was 31.5 ± 17.6 days. The mean body mass index (BMI) was 24.1 ± 2.3 kg/m^2^. Based on pre-injury activity level, 14 patients (66.7%) participated in competitive or recreational sports regularly, while 7 (33.3%) led a sedentary or low-activity lifestyle. The mechanisms of injury were 18 cases due to sports-related trauma (e.g., soccer, basketball, skiing) and 3 due to accidental falls. None of the patients included in this study had concomitant ligament injuries (e.g., PCL, MCL, LCL) or severe meniscal tears such as bucket-handle or root tears. Mild partial meniscal injuries not requiring repair were noted in 4 patients but were not considered exclusionary or clinically significant based on arthroscopic evaluation and patient outcomes. Although quantitative pivot-shift or instrumented rotational laxity measurements (e.g., KT-1000, rotometer) were not available due to the retrospective nature of the study, all included patients demonstrated grade II or III pivot shift under anesthesia, assessed by experienced orthopedic surgeons. This clinical assessment was used as a surrogate for evaluating high-grade rotational instability. This study was approved by the Ethics Committee of the 947th Hospital of the People's Liberation Army of China.

### Inclusion and exclusion criteria

According to the expert consensus on ACL reconstruction and ALL reinforcement or reconstruction established by the ALL Expert Group Meeting in Lyon, France (2015), and the ALL Consensus Group (2018) (9), the medical records included in this study were required to meet the following inclusion criteria: (1) age between 18 and 50 years, with the ability to comply with a 1–2-year postoperative follow-up; (2) confirmed diagnosis of ACL rupture; (3) grade II or III pivot-shift test as determined by specialist examination under anesthesia; (4) no history of previous knee surgery and undergoing knee arthroscopy for the first time; (5) use of the semitendinosus tendon as the graft for ACL reconstruction; (6) presence of generalized joint laxity (Beighton score ≥ 4) or knee hyperextension (>10°); (7) radiological evidence suggestive of a Segond fracture; (8) use of the iliotibial band as the graft for ALL reconstruction. The exclusion criteria were as follows: (1) concomitant injury of other ligaments in the ipsilateral knee; (2) bucket-handle tear of the medial meniscus or root tear of the lateral meniscus on the same side; (3) knee deformity; (4) multiple fractures; (5) abnormal lower limb alignment; (6) neurological disorders or psychiatric illness.

### Surgical methods

#### AIT-ACLR surgical method

**Figure 1:**
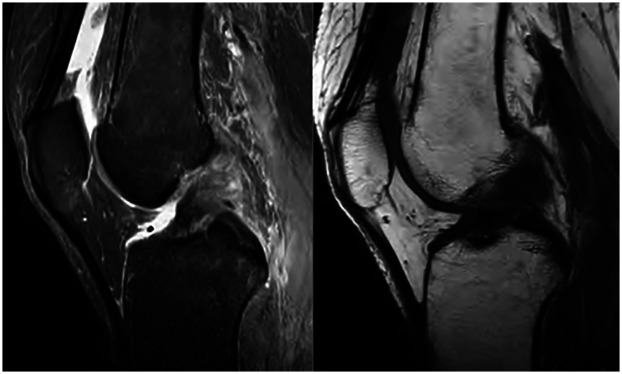
Preoperative MRI images showing anterior cruciate ligament rupture.

All patients in this study underwent an all-inside technique for anterior cruciate ligament reconstruction, and an anterior lateral muscle tendon fixation with the iliotibial band was performed simultaneously, using the semitendinosus tendon as the autograft. The patient was placed in a supine position with spinal anesthesia. No tourniquet was used. Anatomical landmarks, including the patella, patellar ligament, femoral condyle, and tibial plateau, were marked with a marker pen before the operation. The inner and outer approach points were placed with arthroscopy, and the anterior cruciate ligament rupture was confirmed by intra-articular exploration. Graft preparation involved folding the harvested semitendinosus tendon into four strands to improve strength and diameter adequacy. Both ends were whipstitched using high-strength non-absorbable sutures, and fixed with an adjustable loop cortical suspensory fixation device (titanium plate) to ensure secure and reproducible tensioning. Tension was adjusted with the knee in 30° flexion under arthroscopic visualization to avoid over-constraining the joint. This technique offers the advantage of minimizing tunnel length and preserving bone stock while providing adequate initial fixation strength for early rehabilitation. The harvested semitendinosus tendon was cleaned of residual muscle tissue, and folded into quadruple strands to optimize graft thickness and tensile strength. Both ends were whipstitched using a No. 2 non-absorbable braided suture (e.g., Ethibond), ensuring approximately 2 cm of stitched length at each end. Adjustable-loop cortical suspensory fixation devices (TightRope; Arthrex) were then attached to each end, and the graft diameter was measured using a sizing block to ensure optimal tunnel compatibility. Figure 1 illustrates preoperative MRI images, indicating anterior cruciate ligament rupture.

**Figure 2:**
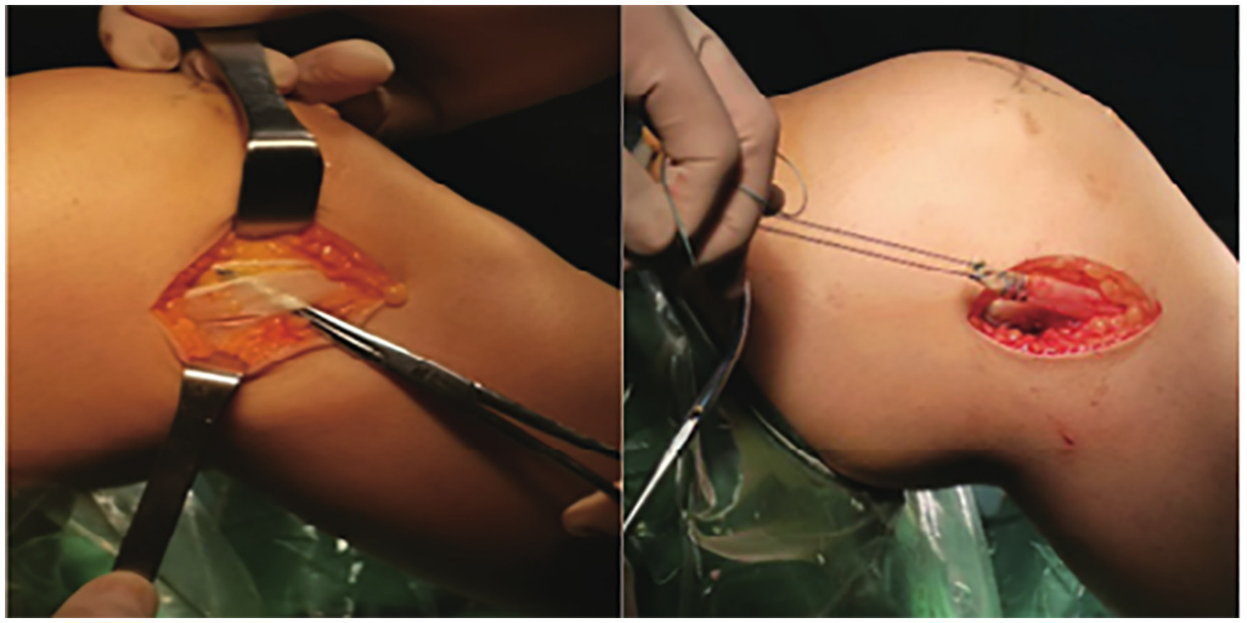
Exposure of the iliotibial band and preparation of anterolateral tendon graft.

AIT-ACLR surgical method: According to the diameter of the prepared graft, a drill bit with the same diameter was used to drill the bone tunnel (Figure 2). The hook was positioned at the tibial end point, and a 4.5 mm flip drill was used to enter the joint cavity. The drill bit was then turned from longitudinal to horizontal, and drilled back from inside the joint cavity to outside the joint, with the length of the bone tunnel restricted to within 3 cm, and at least 5 mm of cortical bone was retained (10). The same method was used to prepare the femoral tunnel. The femoral tunnel was created at the anatomical footprint of the native ACL on the medial wall of the lateral femoral condyle, at approximately the 10 o'clock position in right knees and 2 o'clock in left knees. Tunnel entry was confirmed via arthroscopic visualization to avoid posterior wall blowout. On the tibial side, the tunnel was positioned at the center of the native ACL tibial footprint, just anterior to the medial tibial spine and lateral to the anterior horn of the lateral meniscus, ensuring proper graft alignment and avoiding roof impingement.

The traction lines of the graft at both ends were led out through the tibial and femoral openings, and the locks on both sides of the femur and tibia were tightened and fixed with screws under the stress of posterior tibial pushing with the knee joint flexed at 30°. Femoral and tibial fixation was achieved using the cortical suspensory fixation devices, which were flipped to secure the graft against the lateral femoral cortex and anterior tibial cortex, respectively. After tensioning the graft with the knee in 30° flexion and a posterior drawer force applied to minimize anterior tibial translation, final fixation was performed by securing the adjustable loop with manual traction and knot tying. Tension was reassessed arthroscopically to confirm isometry and graft tautness throughout the range of motion. The tension of the anterior cruciate ligament was checked under arthroscopy to confirm whether there was any impact.

**Figure 3:**
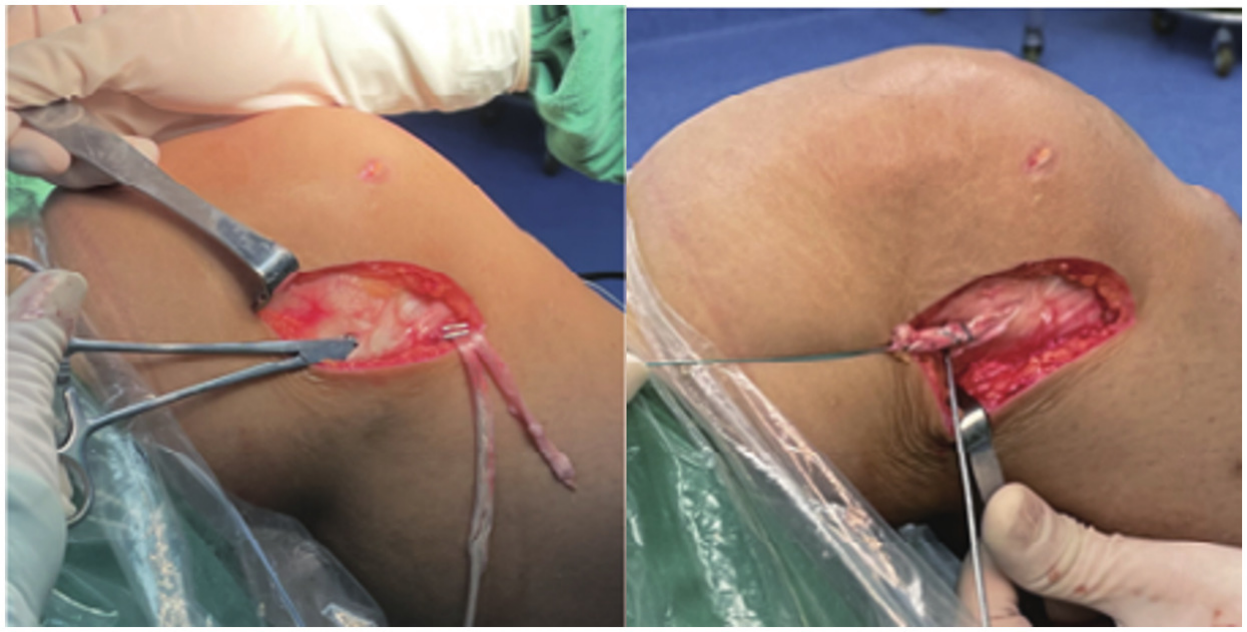
The posterior and upper part of the lateral collateral ligament is the isometric fixation point of ALL tendons.

**Figure 4:**
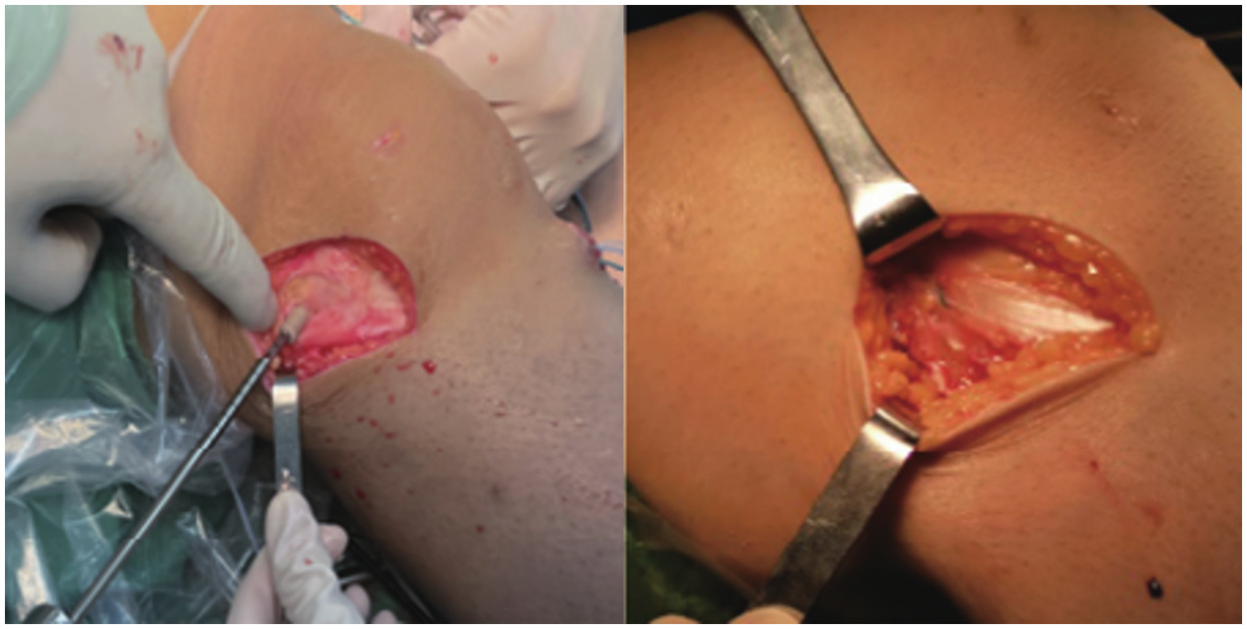
Compression nail fixation after tendon tightening.

**Figure 5:**
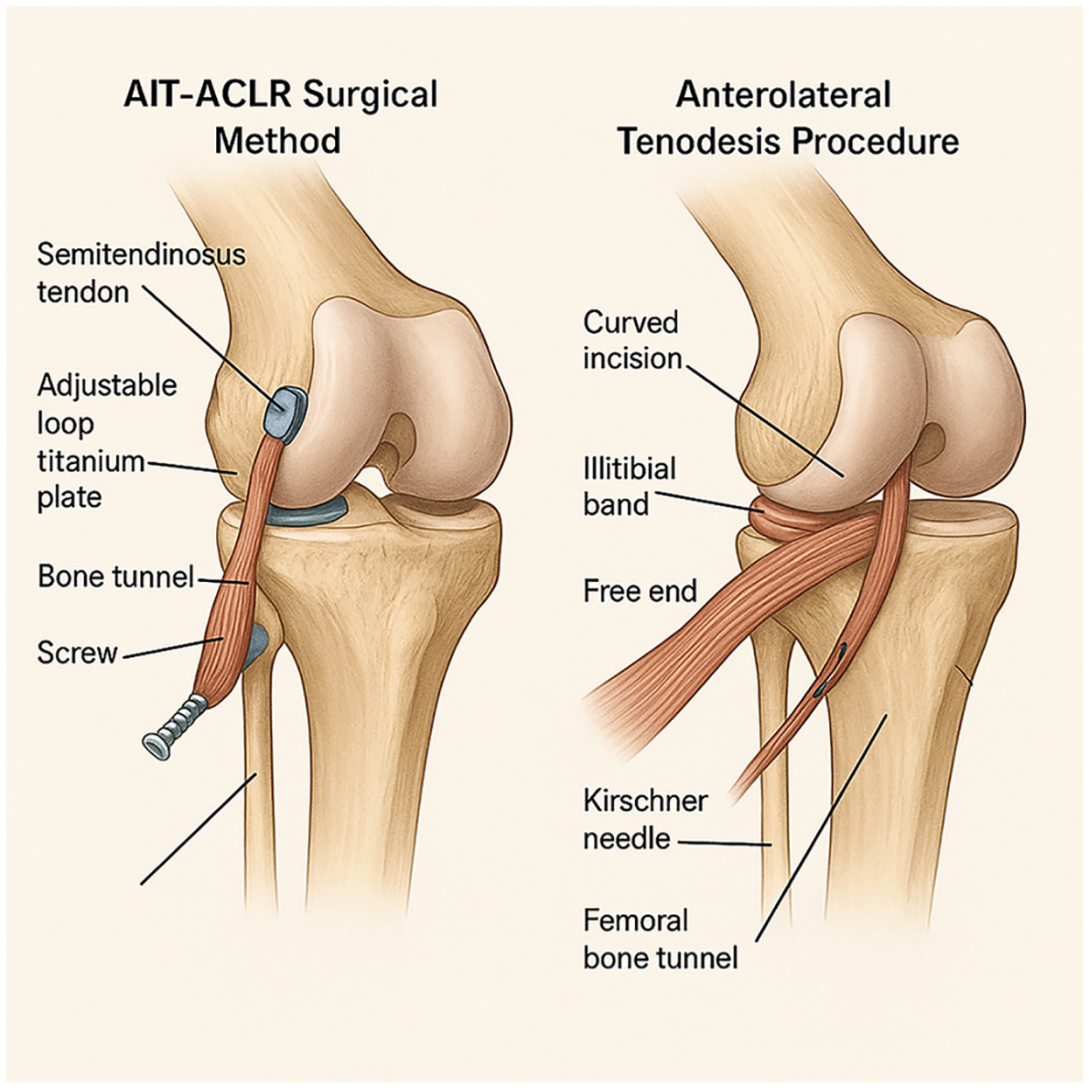
Illustrating surgical techniques, particularly the anterolateral tendon fixation.

Anterolateral tenodesis procedure (ALLT): A curved incision was made from the midpoint between the Gerdy tubercle of the tibia and the head of the fibula to the lateral collateral ligament stop point of the femur. The skin and subcutaneous tissue were cut open, and the iliotibial band was exposed. A 1.5 cm wide tendon was cut from the back edge as the graft, and the distal end was retained at the Gerdy tubercle, separated about 10 cm toward the proximal end, and cut off. The free end of the graft was woven with a suture for about 2.5 cm. The lateral collateral ligament femoral endpoint was exposed, and the same length of fixation point was selected for the ALL tendon. A 2 mm Kirschner needle was inserted into the fixation point, and the free end of the iliotibial band tendon was led through the deep layer of the iliotibial band to the Kirschner needle fixation point (Figure 3). The tendon was passed around the Kirschner needle, appropriately tightened, and the length of the tendon at the end of the weaving was observed at the Kirschner needle fixation point with the knee joint flexed at 0° to 30°. If the length was not satisfactory, another point was selected. After confirming the satisfactory length, a 5–6 mm diameter tunnel was prepared at the Kirschner needle point, and the free end of the woven iliotibial band tendon was led into the femoral bone tunnel and fixed with a screw after tightening (Figure 4). The axial shift test was performed again after fixing the ALL tendon. Figure 5 illustrates the anterolateral tendon fixation.

Among the 21 patients, 2 underwent synovial fold resection on the same side, 3 underwent suture or partial resection of the posterior horn of the medial meniscus on the same side, and 2 underwent resection or suture repair of the anterior horn of the lateral meniscus on the same side. Notably, the fixation point of the ALL graft was slightly proximal and posterior to the anatomical femoral insertion of the native anterolateral ligament. This non-anatomical positioning was selected to achieve better graft isometry and tensioning throughout the range of motion, especially between 0° and 30° of flexion. However, this may alter the physiological biomechanics of the anterolateral structures and potentially increase the risk of over-constraint or limit rotational freedom in some cases. Biomechanical studies have shown that while such non-anatomical tenodesis techniques can improve rotational stability, particularly in high-grade pivot shift knees, care must be taken to avoid excessive internal rotation restraint, which could increase lateral compartment pressure or alter natural joint kinematics. Further long-term studies are needed to assess these implications.

### Postoperative management

**Figure 6:**
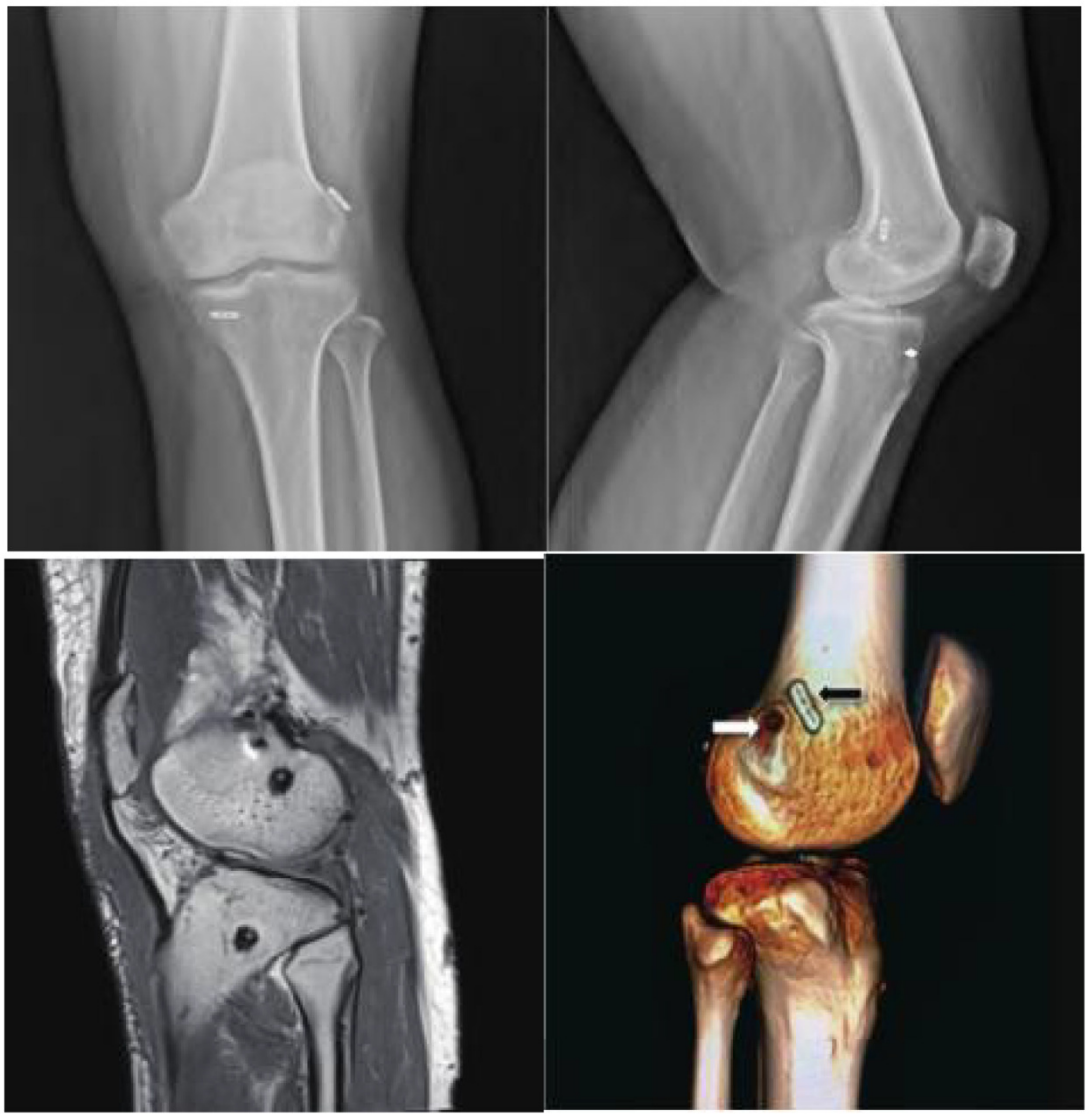
X-ray, MR, and CT images after total internal single beam reconstruction.

After the surgery, routine imaging examination is performed (Figure 6), and symptomatic treatment such as pain relief and anti-inflammatory medication is given. All patients are given support fixation and undergo rehabilitation training. Longitudinal contraction training of the thigh muscles is started on the first day after surgery, and ankle pump training is started on the third day after surgery. Knee joint flexion and extension exercises are performed with gradual weight-bearing under the protection of support fixation after 4 weeks postoperatively, with a requirement of achieving a knee joint range of motion of 120° or higher by the 6th week after surgery. The protective support is removed after 2 months postoperatively, and normal daily activity is achieved between 6 and 8 months after surgery. Sports exercises can be gradually resumed after 1 year postoperatively. Partial weight-bearing with crutches was allowed from postoperative day 1, limited to 20%–30% of body weight, and progressively increased based on patient tolerance and clinical assessment. Full weight-bearing without crutches was generally permitted by 4–6 weeks postoperatively, provided quadriceps control and minimal swelling. For range of motion, passive and active-assisted knee flexion was initiated on postoperative day 2, aiming to achieve 0–90° of flexion by the end of week 2. From weeks 3 to 4, ROM was gradually increased to 120°, with full flexion (135° or more) targeted by 8 weeks. Full extension (0°) was encouraged from the first week and expected by week 3. Use of a hinged knee brace locked in full extension was maintained during ambulation for the first 2 weeks, then gradually unlocked to allow controlled motion as tolerated. High-impact or pivoting activities were strictly avoided for at least 9–12 months.

Return-to-sport (RTS) progression was guided by clinical stability, patient-reported confidence, and functional performance, although specific RTS rates and time frames were not systematically recorded in this study. Based on typical recovery timelines and clinical observations, patients were generally advised to resume gradual sports activities around 12 months postoperatively, contingent on quadriceps strength recovery, knee stability, and absence of effusion or pain. Due to the retrospective design and relatively short follow-up duration, systematic tracking of graft failure rates and complications was not performed. However, no major complications or reoperations were noted during the routine clinical follow-up. Future prospective studies with standardized outcome tracking are warranted to better quantify these important clinical endpoints.

#### Clinical evaluation and statistical analysis

The Lysholm Knee Scoring Scale and International Knee Documentation Committee (IKDC) score were used for subjective evaluation preoperatively and postoperatively. The Lachman test and pivot shift test were used for objective evaluation of anterior and rotational stability of the knee joint. The statistical analysis was performed using SPSS 26.0 software. Continuous variables were expressed as mean ± standard deviation (SD). Paired t-tests were used to compare preoperative and postoperative scores, while the *χ*² test was applied to compare categorical variables such as Lachman test results before and after surgery. In addition, 95% confidence intervals (CIs) were calculated for key outcome measures to assess the precision of estimates. The KT-1000 arthrometer was used to evaluate knee joint stability, and the side-to-side difference between the affected and healthy knee was recorded for objective assessment of recovery. To identify potential predictors of better postoperative outcomes, a multivariate linear regression analysis was conducted. Variables entered into the model included age, sex, time from injury to surgery, presence of meniscal injury, and preoperative IKDC score. *a priori* power analysis was performed using G*Power 3.1.9.7 software to determine the minimum required sample size. Based on an effect size of 0.8, an alpha level of 0.05, and a power of 0.80, the estimated minimum sample size was 15 subjects. Therefore, the final sample of 21 patients was considered sufficient to detect statistically significant differences in primary outcome measures. A *P*-value < 0.05 was considered statistically significant.

## Results

A total of 21 patients (14 men and 7 women) were included in the study, with a mean age of 26.8 ± 6.5 years (range, 18–39 years). The right knee was affected in 12 patients and the left in 9. The average time from injury to surgery was 5.2 ± 2.1 months. The injury mechanism was sports-related in 13 cases, traffic accidents in 5, and falls in 3. Concomitant injuries were observed in 7 patients, including medial meniscus tears in 3, lateral meniscus tears in 2, and synovial fold hypertrophy in 2. The mean BMI of the cohort was 23.4 ± 2.9 kg/m². Notably, 21 patients were followed up for 12 to 24 months with an average follow-up time of (15.6 ± 7.8) months. At the last follow-up, all patients had good recovery without any related postoperative complications.

### Subjective evaluation of knee joint function

#### IKDC score and lysholm score

The preoperative IKDC and Lysholm scores were 48.56 ± 10.33 (95% CI: 43.96 to 53.16) and 52.62 ± 12.41 (95% CI: 46.62 to 58.62), respectively. At the 1-year follow-up, these increased significantly to 86.71 ± 3.62 (95% CI: 85.00 to 88.42) and 89.55 ± 2.87 (95% CI: 88.13 to 90.97), respectively (*P* < 0.01). In addition, at the last follow-up, 11 patients were classified as “excellent” and 8 patients were classified as “good” according to the Lysholm score, which was significantly better than the preoperative classification (*P* < 0.01). These results indicate that the postoperative patients were generally satisfied with the treatment (Table 1).

**Table 1:** IKDC score, lysholm score and lysholm grade of knee joint were compared before surgery and at the last follow-up.

Time	Number of cases	IKDC score ($\bar{x}\pm \text{s}$)	Lysholm score ($\bar{x}\pm \text{s}$)	Lysholm grade (%)			
				Optimal	Good	Normal	Poor
Pre-operation	21	48.56 ± 10.33	52.62 ± 12.41	0	0	15 (71.42%)	6 (28.52)
Last follow-up	21	86.71 ± 3.62	89.55 ± 2.87	11 (52.38%)	8 (38.10%)	2 (9.52%)	0
*t*/*Z*		−13.68	−11.26	−3.59			
*P*		<0.01	<0.01	<0.01			

#### KOOS score

The KOOS score results demonstrated significant postoperative improvements across all subscales. Specifically, the pain score increased from a preoperative value of 28.56 ± 15.85 (95% CI: 21.35–35.77) to 86.36 ± 10.26 (95% CI: 81.69–91.03) at the final follow-up (*P* < 0.01). The symptom score improved from 36.22 ± 12.13 (95% CI: 30.70–41.74) to 73.66 ± 15.87 (95% CI: 66.44–80.88), and stiffness from 62.15 ± 10.58 (95% CI: 57.33–66.97) to 86.89 ± 16.41 (95% CI: 79.42–94.36), both with *P* < 0.01. Similarly, mobility improved from 53.16 ± 11.68 (95% CI: 47.84–58.48) to 81.55 ± 17.28 (95% CI: 73.68–89.42), movement from 23.92 ± 12.56 (95% CI: 18.20–29.64) to 62.11 ± 21.23 (95% CI: 52.45–71.77), and quality of life from 40.33 ± 11.23 (95% CI: 35.22–45.44) to 57.26 ± 16.33 (95% CI: 49.83–64.69), all showing statistically significant differences (*P* < 0.01; Table 2).

**Table 2:** KOOS score before and at the last follow-up ($\bar{x}\pm \text{s}$).

Observation time	Number of cases	Pain	Symptom	Stiffness	Mobility	Movement	Quality of life
Pre-operation	21	28.56 ± 15.85	36.22 ± 12.13	62.15 ± 10.58	53.16 ± 11.68	23.92 ± 12.56	40.33 ± 11.23
Last follow-up	21	86.36 ± 10.26	73.66 ± 15.87	86.89 ± 16.41	81.55 ± 17.28	62.11 ± 21.23	57.26 ± 16.33
*t*		6.21	4.65	5.97	5.98	3.18	8.21
*P*		<0.01	<0.01	<0.01	<0.01	<0.01	<0.01

#### SF-36 scale score

At the final follow-up, SF-36 scores showed improvements in multiple domains. Physiological function increased from 37.89 ± 19.75 (95% CI: 28.90–46.88) to 66.29 ± 25.38 (95% CI: 54.74–77.84), and role-physical from 42.79 ± 12.13 (95% CI: 37.27–48.31) to 59.29 ± 19.37 (95% CI: 50.47–68.11), both with *P* < 0.01. Somatic pain improved significantly from 49.65 ± 20.33 (95% CI: 40.40–58.90) to 88.47 ± 15.91 (95% CI: 81.23–95.71), and general health from 69.76 ± 21.68 (95% CI: 59.89–79.63) to 88.55 ± 9.28 (95% CI: 84.33–92.77). Social function and affective function also improved, from 52.26 ± 18.36 (95% CI: 43.90–60.62) to 78.31 ± 25.82 (95% CI: 66.56–90.06), and from 42.63 ± 16.55 (95% CI: 35.10–50.16) to 71.43 ± 22.15 (95% CI: 61.35–81.51), respectively (all *P* < 0.01). Vitality and mental health scores were maintained with no significant changes; preoperative vitality was 76.93 ± 14.23 (95% CI: 70.45–83.41) and postoperative 81.76 ± 10.15 (95% CI: 77.14–86.38), while mental health was 75.21 ± 18.22 (95% CI: 66.92–83.50) preoperatively and 76.63 ± 17.64 (95% CI: 68.60–84.66) at follow-up (both *P* > 0.05; Table 3).

**Table 3:** SF-36 score before and after last follow-up ($\bar{x}\pm \text{s}$).

Observation time	Number of cases	Physiological function	Physiological function	Somatic pain	General health	Social function	Affective function	Invigoration	Mental health
Pre-operation	21	37.89 ± 19.75	42.79 ± 12.13	49.65 ± 20.33	69.76 ± 21.68	52.26 ± 18.36	42.63 ± 16.55	76.93 ± 14.23	75.21 ± 18.22
Last follow-up	21	66.29 ± 25.38	59.29 ± 19.37	88.47 ± 15.91	88.55 ± 9.28	78.31 ± 25.82	71.43 ± 22.15	81.76 ± 10.15	76.63 ± 17.64
*t*		6.13	7.52	8.46	3.22	5.61	4.33	1.93	0.87
*P*		<0.01	<0.01	<0.01	<0.01	<0.01	<0.01	>0.05	>0.05

#### Predictors of functional outcomes

Multivariate linear regression analysis showed that higher preoperative IKDC scores (*β* = 0.43, *P* = 0.02) and absence of meniscal injury (*β* = −0.39, *P* = 0.03) were independently associated with better postoperative IKDC outcomes. Age, sex, and time from injury to surgery were not significant predictors.

### Objective evaluation of knee joint function

Among the 21 patients, 12 patients (57.14%) had a Lachman test result of grade II and 9 patients (42.86%) had a result of grade III before the surgery. At the 1-year follow-up after the surgery, only 3 patients (14.29%) had a result of grade I, and the remaining 18 patients (85.71%) had negative results, which was significantly different from the preoperative results (*P* < 0.01). In addition, all patients had positive results (grade III) in the pivot shift test before the surgery, but only 2 patients (9.52%) had a result of grade I at the last follow-up, and the remaining patients had good recovery with negative results, which was significantly different from the preoperative results (*P* < 0.01) (Table 4).

**Table 4:** Comparison of the results of lachman test and axial shift test before and at the last follow-up.

Time	Number of cases	Lachman test				Axial displacement test			
		Positive			Negative	Positive			Negative
		Degree I	Degree II	Degree III		Degree I	Degree II	Degree III	
Pre-operation	21	0	12 (57.14%)	9 (42.86%)	0	0	0	21	0
Last follow-up	21	3 (14.29%)	0	0	18 (85.71%)	2 (9.52%)	0	0	19 (90.48%)
÷^2^		47.56				49.12			

## Discussion

In recent years, the incidence of knee joint injuries has been increasing, especially ACL rupture has become a common sports injury. After ACL injury, it will affect the stability and biomechanical balance of the knee joint, and in severe cases, it can involve the meniscus and cartilage, and even cause joint degeneration and osteoarthritis. At present, arthroscopic ACL reconstruction has become an important treatment for ACL injury. However, conventional single-bundle reconstruction requires the use of two tendons, the semitendinosus and gracilis, and has disadvantages such as uneven tension, incomplete bone tunnel filling, and time-consuming and laborious flipping. Studies have shown that the hamstring tendon plays an important role in the knee joint's flexion and internal rotation, and if the hamstring tendon is missing, the knee joint's flexion and internal rotation strength will be reduced by 5% to 10% (11). In addition, the gracilis muscle plays an important role in knee flexion activities over 70°, so preserving the hamstring tendon and the gracilis muscle is particularly important for postoperative rehabilitation (12). This study used the AIT-ACLR technique, which has the following advantages: (1) small skin incision and less bone removal. In the preparation of the bone tunnel at the tibial end, the conventional method is to open the tunnel completely. AIT-ACLR first uses a flip drill to punch through and then turns the drill from longitudinal to transverse and reverse to punch a thick tunnel, which not only helps to preserve the lateral cortical bone of the tibial end, reduce bone removal and surgical incision, but also significantly reduces the incidence of plateau fractures and tunnel ruptures (13). (2) Only the semitendinosus is taken for transplantation, and the gracilis muscle is preserved. The biomechanical characteristics of the ACL are closely related to its cross-sectional area. When the diameter of the graft is less than 8 mm, the risk of surgical failure will increase significantly. Compared with the graft length (11–13) cm required for conventional reconstruction, the AIT-ACLR technique requires only (5–7) cm of graft for ACL reconstruction because of the way the drill is punched through the thick bone tunnel (14). According to the length of the semitendinosus obtained, it can be divided into three or four sections, and its diameter can reach (8–9) mm (15). (3) The bone tunnel is closed and fixed on both sides, with strong stability. Monaco et al. (16) found that compared with the full internal technique of using compression nails to fix the tibial shaft, the conventional single-bundle reconstruction has a risk of tibial tunnel enlargement, compression nail loosening and failure, etc. (4) Light postoperative pain. Because the full internal technique establishes a half tunnel in the femur and tibia, compared with the full bone tunnel and compression nail fixation used in conventional surgery, the damage to the bone cortex and periosteum is smaller, so using the AIT-ACLR technique can significantly reduce postoperative pain.

Considering the variability in femoral attachment points reported in the literature, we selected the fixation point above the posterior fibers of the iliotibial tract at the level of the lateral collateral ligament insertion. This location was chosen to balance graft isometry, minimize iatrogenic injury, reduce incision size, and improve patient tolerance of the procedure. In addition, the fixation technique used for the anterolateral tendon in this study represents a non-anatomical reconstruction method. To date, there are no published clinical studies in China reporting outcomes of combined anterolateral tendon fixation, and it remains uncertain whether this technique may lead to ligament relaxation or failure in the long term. Future studies with larger sample sizes, longer follow-up periods, and imaging-based evaluations such as postoperative MRI or second-look arthroscopy are necessary to validate the durability and biomechanical effectiveness of this approach.

In recent years, the mainstream view is that clinical physicians should pay more attention to the rotational stability of the knee joint while paying attention to the anterior instability caused by ACL injury, such as how to strengthen the lateral structures of the knee joint to improve the efficacy of ACLR treatment (3, 17). Anatomical and biomechanical studies have shown that the probability of the ALL in the human knee joint is 96%, and the slackness of the ALL is highly correlated with rotational instability, mainly manifested as a positive shift test (18). Some believe that the ALL is a composite structure, and adopting surgical methods that strengthen the fixation of the ALL (such as ACLR combined with ALLT) can effectively restore knee joint rotational stability (19, 20). However, there are also opinions that there is still some controversy about whether to choose reconstruction or strengthening fixation for the ALL, and it has been found that ALL strengthening or reconstruction surgery does not significantly improve knee joint rotational stability (21), mainly because the anatomical structure of the ALL is still unclear (22–24). Previous studies have shown that the ALL has different anatomical attachments to the femur and tibia. The controversy about the tibial attachment point of the ALL is small and is generally located between the Gerdy's tubercle and the middle of the fibular head, while the femoral attachment point of the ALL varies greatly. For example, OCKULYAC et al. (24) believed that the femoral attachment point was located at the posterior or upper part of the lateral femoral condyle, while VEREECKE et al. (25) believed that the femoral attachment point of the ALL was slightly anterior to the lateral collateral ligament attachment point. In this study, we comprehensively considered factors such as incision size, iatrogenic injury, ALL length, and patient surgical acceptance, and used the attachment point of the lateral collateral ligament above the posterior fibers of the iliotibial tract as the isometric fixation point of the ALL tendon for anterior lateral tendon fixation.

Recent advances in the understanding of the ALC of the knee, involving the ALL, ITB, and surrounding capsular structures, have underscored its critical role in controlling internal tibial rotation and contributing to pivot shift phenomena (26, 27). Biomechanical studies have demonstrated that isolated ACL reconstruction may be insufficient to fully restore rotational stability in high-demand athletes or patients with high-grade pivot shifts, thereby reinforcing the rationale for augmenting ACL reconstruction with lateral extra-articular procedures (28). Several surgical techniques have been proposed for combined ACL and anterolateral stabilization, including anatomic ALL reconstruction, the modified Lemaire procedure, and ITB-based tenodeses (29). Compared to anatomic ALL reconstruction, which aims to replicate the specific femoral and tibial footprints of the ALL, ITB-based techniques offer technical simplicity and robust biomechanical strength without the need for additional graft harvesting (30). Our approach, using a strip of the ITB for anterolateral reinforcement, aligns with the concept of lateral tenodesis rather than strict anatomical reconstruction, but still offers effective control of rotational laxity. Consistent with the previous findings (31), demonstrating improved pivot shift control with combined ACL + lateral procedures, our results showed substantial improvements in both Lachman and pivot shift tests at final follow-up. The use of the ITB as a graft source for anterolateral augmentation has several advantages: it preserves hamstring tendons, provides a native extra-articular structure with high tensile strength, and allows for a shorter rehabilitation timeline due to minimal donor site morbidity (32). However, potential disadvantages include the risk of over-constraining the lateral compartment if graft tension is excessive, and the non-anatomic nature of the procedure may limit its long-term efficacy in some patients. Future studies comparing different lateral augmentation techniques with long-term follow-up and imaging verification would help determine the optimal strategy for managing combined anterior and rotational instability of the knee.

This study used the AIT-ACLR technique and anterior lateral tendon fixation to treat ACL rupture patients with high-grade shift instability. After surgery, all patients had no complaints of incision infection, knee joint stiffness, pain, etc., and were satisfied with the recovery of knee joint anterior and rotational stability. Considering the variability in femoral attachment points reported in the literature, we selected the fixation point above the posterior fibers of the iliotibial tract at the level of the lateral collateral ligament insertion. This location was chosen to balance graft isometry, minimize iatrogenic injury, reduce incision size, and improve patient tolerance of the procedure. In addition, the fixation technique used for the anterolateral tendon in this study represents a non-anatomical reconstruction method. To date, there are no published clinical studies in China reporting outcomes of combined anterolateral tendon fixation, and it remains uncertain whether this technique may lead to ligament relaxation or failure in the long term. Future studies with larger sample sizes, longer follow-up periods, and imaging-based evaluations such as postoperative MRI or second-look arthroscopy are necessary to validate the durability and biomechanical effectiveness of this approach.

This study has several limitations that must be acknowledged. Firstly, the sample size was relatively small (*n* = 21), which might limit the statistical power and reduce the generalizability of the findings to a broader population. Secondly, the absence of a control group, such as patients treated with standard ACL reconstruction alone, precludes direct comparison and makes it difficult to isolate the specific effect of anterolateral tendon fixation. Additionally, although the follow-up period ranged from 12 to 24 months (mean 15.6 months), this duration is relatively short for orthopedic interventions, particularly when assessing long-term graft durability, functional stability, and complications such as tunnel widening or ligament laxity. Importantly, no patients underwent second-look arthroscopy or follow-up MRI, limiting the ability to evaluate intra-articular healing and graft integrity beyond clinical assessments. Furthermore, the anterolateral tendon fixation technique used in this study represents a non-anatomical reconstruction approach, and its long-term biomechanical and clinical implications remain unclear. There are currently no published clinical reports from China on this type of combined fixation, and whether this technique may lead to ligament relaxation or failure over extended follow-up (e.g., 5–10 years) requires further investigation. Future studies with larger cohorts, randomized controlled designs, imaging-based follow-up, and extended observation periods are necessary to validate the safety, efficacy, and durability of this combined surgical approach.

## Conclusion

In conclusion, in patients with ACL rupture and grade III pivot-shift instability, AIT-ACLR combined with anterolateral tendon fixation could effectively improve both rotational and anterior knee instability, restore joint stability, and possess several advantages, including a small skin incision, minimal bone removal, a closed bone tunnel, bilateral suspensory fixation, preservation of the gracilis tendon, and reduced postoperative pain.

## Data Availability

The original contributions presented in the study are included in the article/Supplementary Material, further inquiries can be directed to the corresponding author.
